# Migrant health penalty: evidence of higher mortality risk among internal migrants in sub-Saharan Africa

**DOI:** 10.1080/16549716.2021.1930655

**Published:** 2021-06-16

**Authors:** Carren Ginsburg, Philippe Bocquier, Ashira Menashe-Oren, Mark A. Collinson

**Affiliations:** a Medical Research Council/Wits Rural Public Health and Health Transitions Research Unit (Agincourt), School of Public Health, Faculty of Health Sciences, University of the Witwatersrand, Johannesburg, South Africa; b Institut d’analyse du changement dans l’histoire et les sociétés contemporaines (IACCHOS), Centre De Recherches En Démographie, Université Catholique De Louvain, Louvain-la-Neuve, Belgium; c Department of Science and Innovation/South African Medical Research Council, South African Population Research Infrastructure Network, South Africa

**Keywords:** Internal migration, mortality, life-course, Africa, health and demographic surveillance system

## Abstract

**Background**: Despite the greater attention given to international migration, internal migration accounts for the majority of movements globally. However, research on the effects of internal migration on health is limited, with this relationship examined predominantly in urban settings among working-age adults, neglecting rural populations and younger and older ages.

**Objectives**: Using longitudinal data from 29 mostly rural sub-Saharan African Health and Demographic Surveillance Systems (HDSS), this study aims to explore life-course differences in mortality according to migration status and duration of residence.

**Methods**Cox proportional hazards models are employed to analyse the relationship between migration and mortality in the 29 HDSS areas. The analytical sample includes 3 836,173 people and the analysis spans 25 years, from 1990 to 2015. We examine the risk of death by sex across five broad age groups (from ages 1 to 80), and consider recent and past in- and return migrants.

**Results**: In-migrants have a higher risk of mortality compared to permanent rural residents, with return migrants at greater risk than in-migrants across all age-groups. Female migrants have lower survival chances than males, with greater variability by age. Risk of dying is highest among recent return migrant females aged 30–59: 1.86 (95% CI 1.69–2.06) times that of permanent residents. Only among males aged 15–29 who move to urban areas is there evidence of a ‘healthy migrant’ effect (HR = 0.62, 95% CI 0.51–0.77). There is clear evidence of an adaptation effect across all ages, with the risk of mortality reducing with duration following migration.

**Conclusions**: Findings suggest that adult internal migrants, particularly females, suffer greater health disadvantages associated with migration. Policy makers should focus on improving migrant’s interface with health services, and support the development of health education and promotion interventions to create awareness of localised health risks for migrants.

## Background

Despite the greater attention given to international migration, internal migration, the movement of people within national boundaries, accounts for the majority of movements globally [1,2]. Migration flows are often between rural and urban areas, and have commonly been explored in relation to urbanisation patterns and processes, in the context of economic development. Nevertheless, rural-rural migration also accounts for a large proportion of internal movement in Low- and Middle-Income Countries (LMIC), often related to agricultural work or to marriage [2–5]. Indeed, the diverse conditions surrounding movement contribute to the complex relationship between migration and health. For example, the health of refugees tends to be poorer than the health of labour migrants [6].

The evidence on the effect of internal migration on health is nevertheless limited, with existing studies contributing mixed evidence. Migration, which results in a change in environment and health exposure, is known to impact health through a range of mechanisms [7,8]. These mechanisms can be broadly categorised as selection, disruption and adaptation [8,9]. Migrants are generally selected in their place of origin as being healthier [10,11], although this ‘healthy migrant’ effect has not been observed among children [12]. Conversely, ‘return’ migrants have been observed as less healthy compared to origin populations, often moving back home in older ages to seek palliative care since they face difficulties in accessing health services in their migration destinations [13,14]. This generates an unhealthy return migrant effect (also referred to as ‘salmon bias’) which leaves healthier migrants at destinations [7].

Disruption due to migration has been explored mostly in relation to reproductive health where migration is associated with lower fertility [15,16]. However, the effects of disruption on health are wider, and may also impact vaccination schedules [17,18]. More broadly, migrating often involves an abrupt change in environment, altering exposures to pollution or malaria for example, which can affect health [19]. Changes in living conditions and social circumstances associated with movement may also have an effect on mental health [20].

In the period following migration, health may be affected through a process of adaptation, or assimilation. For example, relocation from rural to urban settings can result in lifestyle changes with respect to diet and exercise regimes, altering the risk of cardiometabolic disease [21]. Adaptation corresponds to the duration of residence and it has been observed that negative effects of migration on health may be reduced with increased duration of residence as a result of adaptation to the new environment [22,23].

These mechanisms describing the relationship between migration and health will differ depending on a range of individual, environmental and contextual factors. They may also be affected by the stage of the life-course (age) and sex [9]. Indeed migration rates vary by age, as does mortality and causes of death [24]. Among working-age adults, the migration–health relationship is diverse, and influenced by the disease and epidemic dynamics affecting a particular region. Older adult migrants, above age 60, tend to have lower mortality but higher morbidity, though evidence of this in LMICs is limited [20]. In the case of young children, movement may often be prompted by parental migration [25]. Hence selection on health may relate to the health status or circumstances of mothers or accompanying adults [12,26]. Studies of rural-urban migrant children moving together with their mothers have found somewhat lower risk of death when compared with non-migrant rural children, independent of maternal characteristics [27]. To our knowledge, no longitudinal study has systematically explored the relationship between migration and mortality over the life-course across sub-Saharan Africa (SSA).

This study explores the relationship between internal migration and mortality – a major indicator of health – across the life-course. It uses longitudinal data from 29 SSA Health and Demographic Surveillance Systems (HDSS) covering 25 years and 3 836,173 people. SSA provides an opportune setting to examine this relationship as it remains predominantly rural, with relatively high permanent and temporary migration intensities [28,29], in addition to a heavy disease burden of both infectious and non-communicable diseases [30]. The objectives of this study are 1) to explore differences in mortality by in- and return migrant status over the life-course for males and females – previous papers using similar data on fewer HDSS showed higher mortality for migrants than for non-migrants but analysed the 15–59 age group as a whole [22,31], and this needs to be confirmed at a more detailed age granularity, including the often-neglected children aged 5–14 and adults over age 60, and 2) to assess the effect of duration of residence on migrant’s mortality (the adaptation effect) over a wide range of settings across SSA. We anticipate that risk of mortality among migrants, if higher than for non-migrants, should converge to that of non-migrants with longer duration of stays following migration.

## Methods

**Figure 1.**
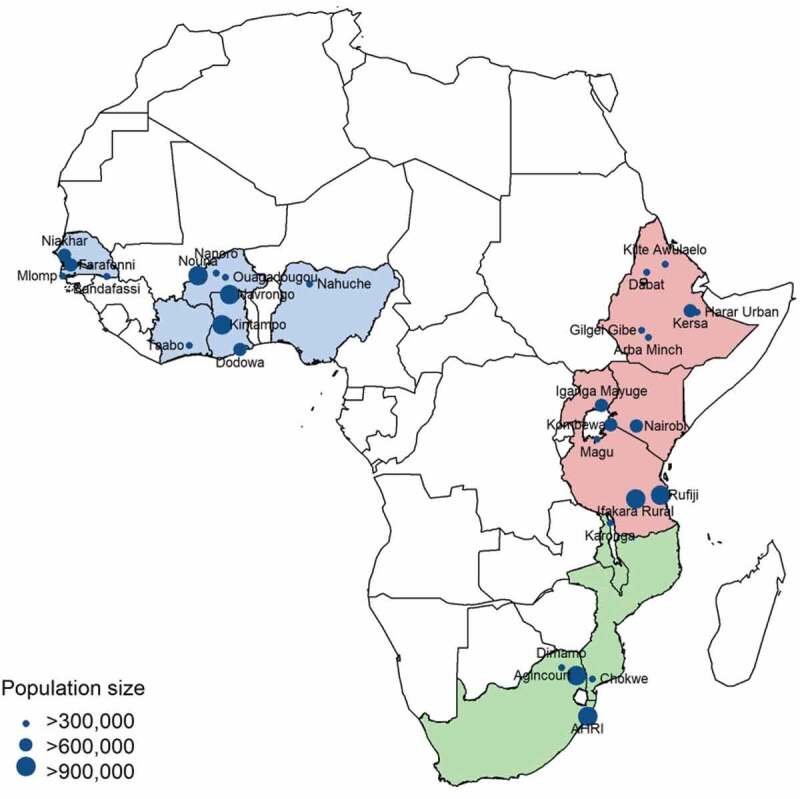
Map of HDSS sites

This study employs data from 29 HDSS in SSA to examine the relationship between internal migration and mortality. HDSS monitor all births, deaths (often including causes of death) and migrations within a geographically defined population over time [32]. The International Network for the Demographic Evaluation of Populations and their Health (INDEPTH: www.indepth-network.org)^1^^1^INDEPTH: www.indepth-network.org brings together 49 HDSS sites in LMICs, 37 of which are located in SSA.^2^^2^The 29 SSA HDSS sites selected for inclusion in the analysis were chosen based on availability of data on iShare, consistency checks and whether the available person-years were sufficient. HDSS provide a valuable source of data, compensating for the lack of comprehensive national civil registration and vital statistics systems in SSA. The locations of the HDSS sites included in the study are presented in Figure 1. We analyse all-cause mortality, by sex and for five broad age groups (1–4 year-olds, 5–14 year-olds, 15–29 year-olds, 30–59 year-olds, and 60–79 year-olds). The analysis excludes infants under 1 year old because differences in mortality among infants in this age-group would necessarily be related to a mother’s migration status, and would be biased in favour of healthier children who are able to move.

Data employed in this study are open access, and available from the INDEPTH iShare platform [33]. The datasets detail all births, deaths and in- and out-migrations with respect to these surveillance populations. This information is collected through regular household visits (at minimum once a year, with frequency varying by HDSS site). Deaths are often confirmed by multiple individuals, and the precise timing of death is verified [32]. Corresponding dates of births and migrations are also captured. The individual-level data allow for the identification of new in-migrants to the HDSS areas, and return migrants (individuals who have lived in the HDSS area for periods exceeding 6 months, and returned following a period away). Across the HDSS sites, a migration may be defined after a period between 3 to 6 months following entry. We adopt the conservative six-month threshold to achieve consistency across sites, and avoid including temporary visitors to HDSS areas as migrants. Permanent residents (or non-migrants) are individuals who have never moved out of the HDSS areas. We also consider in-migrants or return migrants who have lived in the HDSS areas for 10 or more years as permanent residents. We do not examine return migration trends of children under 5 since they have not lived long enough to have moved twice; the same holds for 5 to 14 year-old return migration of durations longer than 2 years following return. To determine adaptation, we examine the length of time since in- or return migration to the HDSS site, whether less than 2 years, between 2 and 5 years or 5 to 9 years. In-migration rates are expressed as the number of in-migration events divided by the person-years of the population at risk (PYAR). The period under consideration in the analysis is from 1990 to 2015, and HDSS sites contribute different periods to the analysis depending on the length of time since inception. All sites contribute data from the year 2012 onwards.

The characteristics of the 29 HDSS sites included in the analysis are presented in Table 1. The majority of the HDSS sites (26 of the 29) are located in rural or mostly rural areas (defined as such on the basis of population density and surroundings). Three of the HDSS sites are urban neighbourhoods, two of which are located in capital cities. The urban HDSS are analysed separately because of the different patterns of in- and return migration encountered with respect to these settlement types. They are included in the study to provide a contrast to the rural perspective presented, given the important links between rural and urban centres within countries represented in these analyses.

**Table 1.** HDSS site characteristics

Site	Country	Start and end of HDSS (from 1 January to 31 December)	Settlement type	Person-years at risk (exposure)	Probability of child death: 5q0	Probability of adult death: 45q15	In-migration rate	Out-migration rate	HIV prevalence as % in adults aged 15–49 (2010) [46]	Gross cell product in US$ at purchase power parity exchange rates (2005) [47]
Nanoro	Burkina Faso	2009–2014	Rural	324,630	0.06	0.20	85.70	126.54	1.0	1.015
Nouna	Burkina Faso	1998–2015	Mostly rural	1 228,023	0.11	0.26	75.72	106.14	0.8	0.182
Ouagadougou	Burkina Faso	2009–2015	Urban	554,584	0.05	0.18	100.54	141.86	1.7	2.132
Taabo	Cote d’Ivoire	2009–2016	Mostly rural	316,338	0.10	0.27	101.76	148.39	3.9	0.944
Gilgel Gibe	Ethiopia	2006–2015	Mostly rural	500,458	0.09	0.24	38.59	58.06	0.5	1.237
Kilte Awulaelo	Ethiopia	2010–2014	Mostly rural	325,211	0.04	0.11	24.72	73.91	1.2	0.553
Kersa	Ethiopia	2008–2016	Mostly rural	625,960	0.10	0.29	10.21	29.60	1.0	0.740
Harar Urban	Ethiopia	2012–2016	Urban	173,915	0.01	0.19	58.26	116.94	1.0	0.740
Dabat	Ethiopia	2009–2015	Mostly rural	343,874	0.03	0.20	32.48	62.94	1.2	0.590
Arba Minch	Ethiopia	2010–2015	Mostly rural	406,654	0.04	0.16	43.00	59.42	1.1	2.029
Navrongo	Ghana	1994–2014	Mostly rural	1 552,935	0.12	0.38	111.37	109.36	1.4	0.111
Kintampo	Ghana	2006–2014	Mostly rural	1 133,005	0.06	0.26	97.62	124.22	1.6	0.275
Dodowa	Ghana	2006–2011	Mostly rural	637,472	0.03	0.29	93.80	131.77	2.3	1.222
Farafenni	Gambia	1990–2015	Mostly rural	773,793	0.09	0.26	81.95	106.89	1.8	1.306
Nairobi	Kenya	2003–2015	Urban	770,322	0.07	0.33	199.37	270.16	6.2	2.502
Kombewa	Kenya	2011–2015	Rural	649,740	0.06	0.30	76.11	93.83	4.4	4.386
Karonga	Malawi	2003–2016	Mostly rural	472,001	0.06	0.30	78.03	104.25	9.0	0.360
Chokwe	Mozambique	2010–2015	Mostly rural	461,903	0.07	0.40	66.76	123.63	25.8	0.473
Nahuche	Nigeria	2011–2014	Rural	526,069	0.27	0.26	7.90	24.40	1.4	1.996
Bandafassi	Senegal	1990–2016	Rural	300,114	0.18	0.29	35.51	23.01	1.4	0.073
Mlomp	Senegal	1990–2016	Rural	210,522	0.08	0.22	76.14	77.42	1.6	0.559
Niakhar	Senegal	1990–2016	Rural	880,616	0.12	0.22	56.61	49.83	0.4	1.306
Ifakara Rural	Tanzania	1997–2014	Rural	1 502,326	0.10	0.28	102.47	158.80	5.3	0.476
Rufiji	Tanzania	1999–2014	Mostly rural	1 229,392	0.09	0.28	74.62	115.20	4.6	1.176
Magu	Tanzania	1994–2013	Rural	446,678	0.10	0.37	167.17	211.72	5.8	0.816
Iganga/Mayuge	Uganda	2005–2015	Mostly rural	681,290	0.09	0.23	92.07	127.37	4.8	3.017
Agincourt	South Africa	1993–2016	Rural	1 950,892	0.05	0.38	61.18	90.29	22.3	1.070
Dimamo	South Africa	1996–2016	Rural	362,059	0.02	0.33	31.04	40.13	12.9	3.400
AHRI	South Africa	2000–2016	Mostly rural	1 142,236	0.06	0.54	84.83	122.35	26.1	0.334

Analyses are conducted on pooled data from all sites using Cox proportional hazards models. The Cox model combines survival analysis with regression analysis to investigate the effect of a set of covariates on the risk of experiencing an event, in our study death. Models control for site-period effects as there may be high variation in levels of migration at different times and because mortality may differ by site and over different periods. All analyses are performed separately for males and females, and by discrete age groups, to account for any differences in the migration–mortality relationship between the sexes or over the life-course. Analyses are conducted separately for the urban and rural sites.

## Results

### Descriptive results

**Figure 2.**
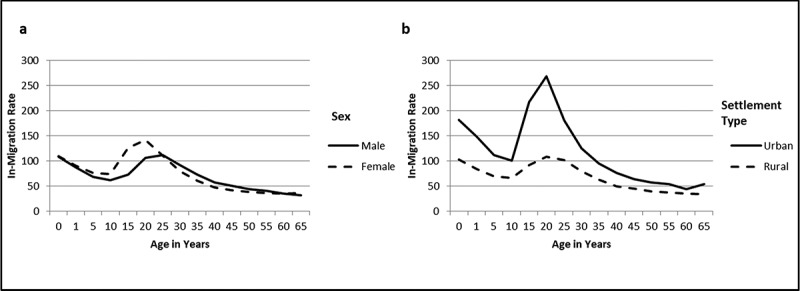
(a, b) In-migration patterns by sex and settlement type^1^

^1^In-migration patterns differ significantly by sex (HR = 1.13, 95% CI 1.12–1.13) and settlement type (HR = 0.48, 95% CI 0.48–0.49).

The in- and out-migration rates, presented by site for the period of analysis in Table 1, show considerable diversity between sites. In nearly all sites, out-migration exceeds in-migration. In-migration rates across all sites reach a peak in early adult years (ages 15–29) for both males and females, in both rural and urban settlement types (Figure 2(a). In-migration into these mostly rural local areas is more prevalent in females in early adult ages (141.23 95% CI 140.26–142.00) per 1000 PYAR among 20–25 year-olds) who are likely relocating following marriage, or for family reasons. The urban migration destinations exhibit far higher rates of in-migration particularly in working-age adults individuals. Between ages 20 and 25 rates of in-migration to urban sites are 267.82 (95% CI 265.45–270.21) per 1000 PYAR.

**Figure 3.**
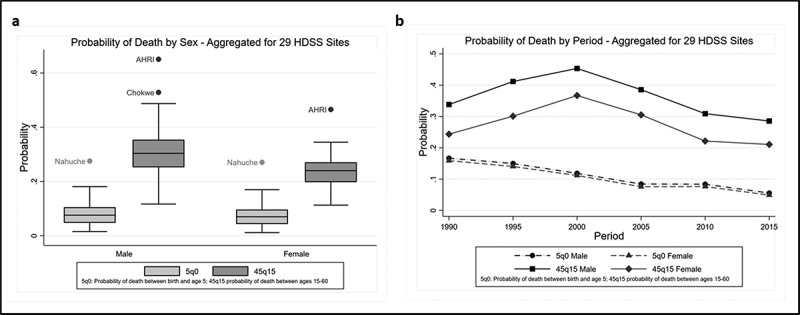
(a, b) Mortality patterns by sex and period

Mortality patterns for the 29 sites in the pooled sample are presented by sex and period in Figure 3a and b. Mortality is represented as the probability of death in children under 5 years (_5_q_0_) and among adults aged between 15 and 60 years (_45_q_15_, premature adult mortality), two commonly used indicators of population-level mortality. The median probability of premature adult mortality among males in the sample is 0.30 (interquartile range [IQR] 0.25–0.35) and in females 0.24 (IQR 0.20–0.27). The AHRI in South Africa and Chokwe in Mozambique have outlying probabilities of premature adult mortality (ARHI males = 0.65, females = 0.47; Chokwe males = 0.53), while Nanuche in Nigeria has outlying probabilities of under 5 mortality in males (0.28) and females (0.27). Period trends in the probability of mortality are consistent for both sexes but differ between children and adults. Among children mortality has steadily declined, while for adults mortality increased and then declined. The increasing probability of premature mortality in adults leading up to the year 2000 likely captures the rising HIV epidemic, mainly in Southern and Eastern African HDSS (see Table 1), which decreases following the roll-out of antiretroviral therapy. This is consistent with mortality trends for SSA as a whole, with Southern Africa displaying the highest rates of adult mortality over the past two decades, and mortality declining across SSA from around 2005 to present [34]. Although the mortality estimates are not nationally representative, the sites are sufficiently diverse to capture the broad patterns of mortality across SSA.

### Results of the Cox models

**Figure 4.**
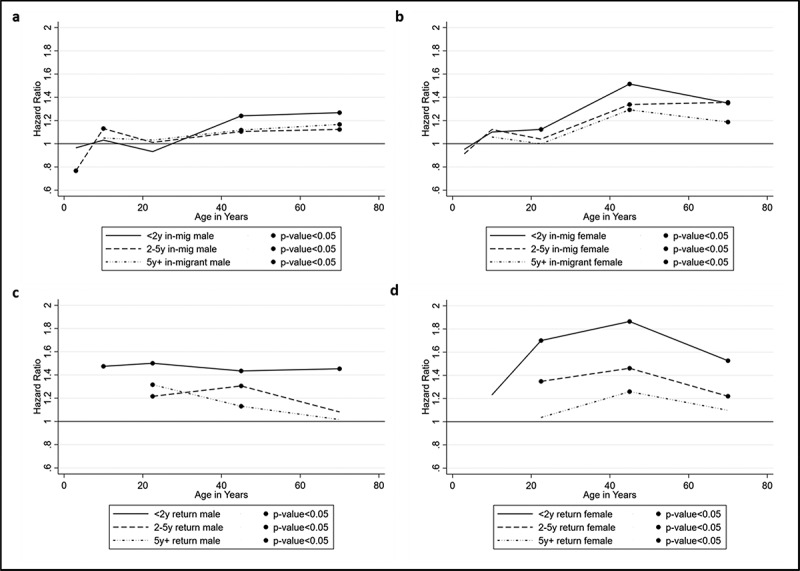
(a–d) Effect of migration status by duration since migration by age and sex: rural HDSS sites

**Figure 5.**
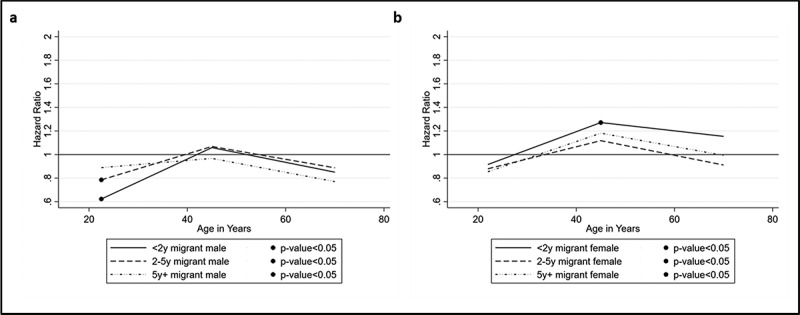
(a ,b) Effect of migration status by duration since migration by age (15–8) and sex: urban HDSS sites

The results of the Cox proportional hazards models are presented as hazard ratios (HR) in Figure 4a–d and Figure 5a–b (see supplementary material: Appendix 1 and 2 for the full models). The trends for in-migrants to rural HDSS (Figure 4(a) suggest a higher risk of mortality compared with permanent residents across the life-course for females, while for males, there is some variability across the life-course with the trend of higher risk presenting from age 30 onwards. Male in-migrants between ages 30 and 59 have 1.24 (95% CI 1.17–1.31) times higher risk of mortality than permanent residents within the first two years following migration. This higher risk is also significant among males aged 60–79 (HR = 1.27, 95% CI 1.16–1.38). For female in-migrants within the first two years, mortality risk increases over the life-course with hazard ratios of 1.12 (95% CI 1.04–1.21) in the 15–29 age group, increasing to 1.51 (95% CI 1.43–1.61) in 30–59 age group and remaining significantly higher in the oldest age group (HR = 1.35, 95% CI 1.25–1.46).

For children under 15, the impact varies by sex. Mortality risk for boys is less consistent, with significantly lower risk among in-migrant boys under 5 years, and higher risk of mortality among boys aged 5 to 14 years relative to permanent residents (HR = 1.13, 95% CI 1.01–1.26, for those between 2 to 4 years following in-migration). The differences by migrant status in girls under age 15 are not significant when contrasted with permanent residents. Lack of clear trends for children might be indicative of the complex interactions between parent and child migrations in relation to health. For adults, there is clear evidence of an adaptation effect among both male and female in-migrants, with the risk of mortality reducing with duration following migration.

Compared to in-migrants, return migrants have a higher risk of mortality than permanent residents, in particular within the first two years following return to the HDSS (Figure 4(c). Male return migrants have a reasonably consistent higher risk of mortality over the life-course, with the highest risk evident in the younger adult age group (15–29) (HR = 1.50, 95% CI 1.32–1.71), and only slightly lower in the oldest age group of 60–79 (HR = 1.45, 95% CI 1.24–1.70). For females, mortality risk in the two years following return to the HDSS is higher than for males, and significantly higher than permanent residents: 1.70 (95% CI 1.52–1.91) times higher risk in ages 15–29, 1.86 (95% CI 1.69–2.06) times higher risk in the 30–59 age group and 1.53 (95% CI 1.33–1.74) times higher risk in the oldest age group of 60–79. There is once again evidence of an adaptation effect with risk of mortality following return migration declining after 2 years spent in the HDSS. For males aged 15 to 59, risk of mortality declines but remains significantly higher than permanent residents 2–4 years following their return. Similarly for females over age 30, the risk of mortality decreases between 2 and 4 years following return, but remains significantly higher than for permanent residents.

We compared urban HDSS to rural sites, to provide a contrasting perspective. Variations by sub-region were also explored but no significant grouping by West, East or Southern Africa were identified (results not shown). Due to a smaller number of urban sites (only three) and of return migrants, in- and return migrants are collapsed in the urban analysis and age is limited to 15 years and older (Figure 5(a). In contrast with migrants entering rural areas, males aged 15 to 29 years have 0.62 (95% CI 0.51–0.77) times the risk of mortality compared to permanent urban residents. The trend for females suggests higher mortality risk in the 30 to 59 year age group only – female migrants of these ages have 1.27 (95% CI 1.06–1.52) times the risk of mortality as compared with permanent residents, and this risk reduces with longer durations spent in the HDSS.

## Discussion

The analyses presented in this study offer new insights into the internal migration and health relationship over the life-course. The study uses temporally detailed longitudinal data from a range of SSA local areas in the West, East and South of the continent, and offers a unique perspective by focusing on rural areas that receive new in-migrants as well as return migrants. The rural perspective is often overlooked in the literature with emphasis frequently placed on rural-to-urban migration and urbanisation dynamics. These data reinforce the understanding that a substantial number of people are moving between rural areas, or returning home following periods of residence, mostly in urban areas.

The findings provide evidence of health challenges faced by migrants. Both in- and return migrants have a health disadvantage compared with permanent residents of these HDSS areas. This is contrary to what has generally been observed about the health status of international migrants compared to non-migrants in destination countries [35]. Therefore, the ‘healthy migrant effect’ may not apply to all internal migrants in SSA settings, particularly in adult age-groups, except maybe for males moving to urban areas. In rural areas the risk among in-migrants is lower than that of return migrants, likely because in-migrants are coming from other rural areas with similar environmental exposures. In contrast, return migrants have commonly had exposure to urban areas where they have worked away from home and their return may be suggestive of instances of ‘failed’ migration, or unhealthy return migration [13].

Another notable finding of the study is the markedly higher mortality risk by migrant status observed among females compared with males. This more severe unhealthy migrant effect for females suggests they face greater health challenges in their migration compared to males. This is likely associated with the circumstances surrounding the migration. For in-migrants, males more commonly move for reasons related to environmental or economic circumstances, while female migrants are more likely to move in response to social circumstances such as marriage, and are possibly moving together with children [36,37]. Therefore, males may benefit economically from movement, while females may have fewer or more constrained choices in situations surrounding their movements, which may not yield improved socioeconomic benefits. For return migrants who likely moved out of the HDSS areas for reasons associated with employment, females may be more vulnerable to conditions in destination areas, leading to their higher mortality risk on return home [38].

For both sexes, risk of mortality among adult migrants reduces over time. This is consistent with other studies that have identified a positive effect on health with increased duration of residence following internal migration [18,22]. The universality of this adaptation may be interpreted in two ways. Firstly, the convergence of migrant mortality risk with non-migrants may be the result of a selection of survivors over time; and secondly, it can reflect the longer time it takes for migrants to access and (re-) adapt to local health systems. This firm evidence of higher mortality in the first years after migration highlights the need to focus health interventions in periods following migrants’ entry or re-entry into a rural area. This is particularly imperative in cases of return migration, where possible challenges in accessing health services at migrant destinations, or interruptions in continuity of health care for chronic conditions may put people at even higher health risk. A further recommendation for public health intervention relates to health promotion before migration. This may be achieved through creating awareness among prospective migrants of the health risks associated with the migration process itself (e.g. road accidents), and possible exposures at the particular destinations (e.g. working conditions, contagious diseases, diet, and social risks including violence, substance abuse).

While research has commonly focused on working-age adults, this study contributes an analysis of different kinds of migration effects over the life-course. Mortality risks tend to be lower among migrants of younger ages (under age 30), when the likelihood of migration is higher, and higher in mid-adulthood, while migration rates are reduced. Younger adult in-migrants may be more resilient than older in-migrants. In contrast, return migrants experience higher mortality than non-migrants in young and mid-adult ages, decreasing in older ages among females but remaining fairly stable into old age among males. The patterns found in children are less consistent and suggest that circumstances surrounding children’s movements – whether connected to parents’ migrations, or unaccompanied in relation to school or fostering – may play a role in the migration and health relationship. The importance of maternal, household and community characteristics was observed by Mberu and Mutua [39] in their study on migration and mortality in 5–15 year-olds in Nigeria and Kenya. The authors also found that mortality risk in under 15 year-olds differed by country depending on the direction of the migration stream [39]. The diversity of findings highlights the need for future research to extend investigations on the impact of migration on health among children and adolescents.

The urban data present a juxtaposition of the rural perspective, although we recognise the limitations of the small sample of sites. These urban areas are heterogeneous and trends may be driven by context-specific differences. Nevertheless, consistent with the findings for rural areas, the results identify health disadvantages among female migrants, which reduce with duration following a move. For males, there is evidence of positive selection on health in early adult ages (15–29), and this is the only case where this study confirms the ‘healthy migrant’ hypothesis. To note, this does not show for females of the same age-group. Positive selection on health for rural-urban migrants was previously observed in Malawi among adults of both sexes, but in females the effect was reduced when controlling for age [40,41], highlighting the possible divergence in health selection trends by age and sex.

The results of this study contribute valuable evidence of higher mortality among internal migrants in SSA using longitudinal data on an exceptionally large number of individuals (and PYARs) from a range of mostly rural settings. Nevertheless, a few limitations should be noted. The HDSS data do not include information on reasons for moves, or details of in-migrant’s places of origin/return migrant’s locations while away from the HDSS. Thus the specific circumstances surrounding movement and corresponding changes in environmental conditions and exposures while out of the HDSS areas cannot be examined. Further, deaths that occur among out-migrants are not documented: their death risk is measured in the HDSS conditional on their return. Therefore the analysis does not consider mortality risk among migrants who remain outside the HDSS areas. Further, the HDSS sites included in this analysis are not nationally representative, rather they are illustrative of similar sub-district populations of the respective countries. That said, the study examines migration status effects that are present after controlling for contextual variation (heterogeneity) across sites. Nevertheless, additional country- or region-specific analyses that extend the models to include contextual and site-specific socioeconomic indicators would deepen our understanding of the observed migration and health dynamics.

## Conclusion

There is an urgent need for new evidence to enhance current understandings of migration and health dynamics for policy makers, development agencies and the research community [20,42,43]. Strengthening knowledge on internal migration and LMIC contexts is particularly crucial [44]. The 2030 Sustainable Development Goal Three to ‘Ensure healthy lives and promote well-being for all at all ages’ and corresponding efforts towards universal health coverage, motivates for greater attention to be placed on vulnerable groups, women and children [45]. This study on internal migration contributes to this endeavour, showing potential health penalties at different stages of the life-course associated with internal mobility. The findings suggest that internal migrants, and females aged 30–59 in particular, suffer greater health disadvantages. They, more so than non-migrants, are ‘left-behind’ by health systems, reinforcing the need for plans towards universal health coverage to incorporate mobile populations. The findings further highlight potential differences in public health service needs for international and internal migrants, and argue that migration streams to rural areas should not be neglected in policy. This study’s findings can provide SSA policy makers with supporting evidence for the development of health education and promotion interventions for migrants, to create awareness of localised health issues. Such interventions should further focus on health service capacity development in under-resourced rural areas, and improving migrant’s interface with health services.

## Supplementary Material

Supplemental Material

## Data Availability

Data employed in this study are open access, and available from the International Network for the Demographic Evaluation of Populations and Their Health (INDEPTH) iShare platform (http://www.indepth-ishare.org/index.php/home). The original pooled data are available at https://doi.org/10.7796/HDSS.29.SUB.SAHARAN.AFRICA.V1. Stata programs for data shaping and analysis are available at https://github.com/bocquier/mighealth.git.

## Paper Context

Studies on international migration have identified a mortality advantage among migrants, yet research on internal migration and health, over the life-course, is limited. Using data from 29 sub-Saharan African HDSS we focus on migration to rural areas and find that adult female migrants in particular face a mortality disadvantage. Rural migration streams, and corresponding health risks by sex and over the life-course should be accounted for in public health policy.
