# Supplementary material for: Migrant health penalty: evidence of higher mortality risk among internal migrants in sub-Saharan Africa
Source: Glob Health Action. 2021 Jun 16;14(1):1930655. doi: 10.1080/16549716.2021.1930655 (PMC8550177; doi:10.1080/16549716.2021.1930655)
Supplement: Supplemental Material [file ZGHA_A_1930655_SM8471.docx]

**Supplementary Material: Appendix 1: Cox proportional hazards models: Rural HDSS**

| **Variables** |  | **Age 1-4 mortality males** | **Age 1-4 mortality females** | **Age 5-14 mortality males** | **Age 5-14 mortality females** | **Age 15-29 mortality males** | **Age 15-29 mortality females** | **Age 30-59 mortality males** | **Age 30-59 mortality females** | **Age 60-79 mortality males** | **Age 60-79 mortality females** |
| --- | --- | --- | --- | --- | --- | --- | --- | --- | --- | --- | --- |
| **Centre period variables** |  |  |  |  |  |  |  |  |  |  |  |
| Nanoro | 2005 | 1.43* | 1.46* | 1.47 | 0.98 | 1.15 | 0.84 | 1.52* | 1.21 | 0.74 | 0.39*** |
|  |  | (0.96 - 2.13) | (0.94 - 2.27) | (0.74 - 2.92) | (0.43 - 2.23) | (0.49 - 2.67) | (0.36 - 2.00) | (0.97 - 2.38) | (0.80 - 1.81) | (0.51 - 1.07) | (0.23 - 0.65) |
| Nanoro | 2010 | 1.04 | 1.13 | 1.56** | 1.42* | 1.11 | 1.28 | 1.99*** | 0.81 | 0.91 | 0.73*** |
|  |  | (0.81 - 1.33) | (0.86 - 1.48) | (1.04 - 2.35) | (0.94 - 2.15) | (0.73 - 1.68) | (0.85 - 1.92) | (1.58 - 2.50) | (0.63 - 1.05) | (0.76 - 1.09) | (0.60 - 0.89) |
| Nouna | 1995 | 4.16*** | 5.70*** | 4.47*** | 4.94*** | 2.27*** | 3.82*** | 2.67*** | 2.16*** | 1.79*** | 1.99*** |
|  |  | (3.21 - 5.41) | (4.33 - 7.51) | (2.83 - 7.05) | (3.14 - 7.75) | (1.46 - 3.53) | (2.44 - 6.00) | (2.02 - 3.53) | (1.58 - 2.94) | (1.42 - 2.26) | (1.55 - 2.55) |
| Nouna | 2000 | 3.11*** | 3.35*** | 2.33*** | 2.17*** | 1.41** | 2.68*** | 1.94*** | 1.83*** | 1.23** | 1.70*** |
|  |  | (2.52 - 3.83) | (2.66 - 4.21) | (1.57 - 3.46) | (1.45 - 3.24) | (1.01 - 1.96) | (1.86 - 3.85) | (1.57 - 2.41) | (1.46 - 2.30) | (1.03 - 1.46) | (1.43 - 2.03) |
| Nouna | 2005 | 2.72*** | 2.70*** | 1.97*** | 1.81*** | 1.09 | 1.68*** | 1.71*** | 1.41*** | 1.33*** | 1.27*** |
|  |  | (2.21 - 3.34) | (2.15 - 3.40) | (1.33 - 2.91) | (1.22 - 2.70) | (0.78 - 1.51) | (1.16 - 2.44) | (1.38 - 2.12) | (1.12 - 1.77) | (1.12 - 1.57) | (1.06 - 1.51) |
| Nouna | 2010 | 1.97*** | 2.00*** | 1.53** | 1.25 | 0.83 | 1.23 | 1.34*** | 0.97 | 1.11 | 1.15 |
|  |  | (1.59 - 2.42) | (1.59 - 2.52) | (1.03 - 2.29) | (0.83 - 1.88) | (0.59 - 1.16) | (0.84 - 1.80) | (1.08 - 1.66) | (0.77 - 1.23) | (0.94 - 1.32) | (0.96 - 1.36) |
| Nouna | 2015 | 1.94*** | 2.01*** | 1.25 | 0.74 | 0.59* | 0.76 | 1.15 | 1.28 | 1.31** | 1.25* |
|  |  | (1.48 - 2.56) | (1.49 - 2.71) | (0.70 - 2.23) | (0.37 - 1.48) | (0.33 - 1.05) | (0.41 - 1.41) | (0.85 - 1.56) | (0.94 - 1.75) | (1.03 - 1.67) | (0.97 - 1.59) |
| Taabo | 2005 | 1.93*** | 2.74*** | 2.81*** | 1.90 | 1.51 | 1.90* | 2.53*** | 2.02*** | 1.23 | 0.88 |
|  |  | (1.26 - 2.95) | (1.82 - 4.12) | (1.47 - 5.36) | (0.87 - 4.12) | (0.79 - 2.89) | (0.92 - 3.93) | (1.80 - 3.56) | (1.32 - 3.08) | (0.83 - 1.83) | (0.50 - 1.54) |
| Taabo | 2010 | 2.10*** | 2.18*** | 1.95*** | 1.72** | 1.40* | 1.78*** | 1.81*** | 1.68*** | 1.01 | 1.07 |
|  |  | (1.67 - 2.65) | (1.69 - 2.81) | (1.27 - 2.99) | (1.11 - 2.67) | (0.98 - 2.02) | (1.18 - 2.68) | (1.45 - 2.27) | (1.31 - 2.15) | (0.82 - 1.24) | (0.85 - 1.35) |
| Taabo | 2015 | 1.48** | 1.97*** | 1.94** | 1.03 | 1.26 | 1.74** | 1.68*** | 1.21 | 1.07 | 0.98 |
|  |  | (1.09 - 2.01) | (1.44 - 2.68) | (1.16 - 3.24) | (0.55 - 1.93) | (0.78 - 2.02) | (1.05 - 2.87) | (1.29 - 2.19) | (0.88 - 1.66) | (0.82 - 1.39) | (0.71 - 1.35) |
| Gilgel Gibe | 2005 | 1.28* | 1.63*** | 2.34*** | 2.33*** | 1.47** | 2.63*** | 1.50*** | 1.81*** | 0.83 | 1.71*** |
|  |  | (0.99 - 1.66) | (1.24 - 2.14) | (1.54 - 3.54) | (1.54 - 3.52) | (1.01 - 2.13) | (1.79 - 3.84) | (1.17 - 1.93) | (1.40 - 2.34) | (0.65 - 1.05) | (1.36 - 2.16) |
| Gilgel Gibe | 2010 | 0.83 | 1.02 | 1.97*** | 1.40 | 0.75 | 1.66** | 1.27** | 1.36** | 0.75*** | 1.54*** |
|  |  | (0.64 - 1.08) | (0.77 - 1.35) | (1.32 - 2.96) | (0.92 - 2.14) | (0.50 - 1.12) | (1.13 - 2.45) | (1.01 - 1.61) | (1.07 - 1.73) | (0.61 - 0.93) | (1.25 - 1.89) |
| Gilgel Gibe | 2015 | 0.83 | 0.60* | 1.05 | 1.56 | 1.51 | 0.80 | 0.85 | 1.21 | 0.70* | 1.47** |
|  |  | (0.53 - 1.30) | (0.33 - 1.07) | (0.52 - 2.13) | (0.84 - 2.87) | (0.90 - 2.52) | (0.39 - 1.66) | (0.56 - 1.29) | (0.83 - 1.77) | (0.48 - 1.02) | (1.06 - 2.05) |
|  |  |  |  |  |  |  |  |  |  |  |  |
| Kilte Awulaelo | 2010 | 0.44*** | 0.45*** | 1.03 | 0.62* | 0.94 | 0.75 | 0.61*** | 0.70*** | 0.44*** | 0.72*** |
|  |  | (0.31 - 0.62) | (0.30 - 0.65) | (0.67 - 1.60) | (0.38 - 1.01) | (0.66 - 1.34) | (0.48 - 1.15) | (0.47 - 0.80) | (0.54 - 0.91) | (0.36 - 0.53) | (0.60 - 0.88) |
| Kersa | 2005 | 1.59*** | 2.14*** | 2.24*** | 3.02*** | 2.66*** | 2.84*** | 2.44*** | 1.60*** | 1.13 | 1.63*** |
|  |  | (1.21 - 2.09) | (1.61 - 2.85) | (1.41 - 3.56) | (1.95 - 4.69) | (1.83 - 3.85) | (1.87 - 4.33) | (1.90 - 3.12) | (1.19 - 2.15) | (0.86 - 1.50) | (1.24 - 2.15) |
| Kersa | 2010 | 1.87*** | 2.84*** | 2.81*** | 2.71*** | 1.82*** | 1.95*** | 2.05*** | 1.55*** | 1.18* | 1.65*** |
|  |  | (1.49 - 2.34) | (2.25 - 3.60) | (1.91 - 4.12) | (1.84 - 3.98) | (1.32 - 2.50) | (1.34 - 2.84) | (1.66 - 2.54) | (1.23 - 1.96) | (0.97 - 1.43) | (1.36 - 2.01) |
| Kersa | 2015 | 1.29** | 1.60*** | 1.43 | 1.67** | 1.43** | 1.49* | 1.86*** | 1.00 | 0.95 | 1.15 |
|  |  | (1.01 - 1.66) | (1.23 - 2.09) | (0.93 - 2.21) | (1.09 - 2.55) | (1.01 - 2.03) | (0.99 - 2.24) | (1.49 - 2.32) | (0.77 - 1.30) | (0.77 - 1.17) | (0.92 - 1.43) |
| Dabat | 2005 | 0.49** | 0.65 | 1.52 | 0.68 | 1.45 | 2.11*** | 1.72*** | 1.84*** | 0.85 | 1.18 |
|  |  | (0.26 - 0.91) | (0.36 - 1.19) | (0.76 - 3.01) | (0.26 - 1.74) | (0.82 - 2.57) | (1.20 - 3.71) | (1.20 - 2.47) | (1.28 - 2.66) | (0.60 - 1.22) | (0.81 - 1.73) |
| Dabat | 2010 | 0.48*** | 0.58*** | 0.97 | 0.67 | 1.11 | 1.50** | 1.03 | 1.14 | 0.62*** | 1.36*** |
|  |  | (0.34 - 0.67) | (0.41 - 0.83) | (0.60 - 1.55) | (0.40 - 1.13) | (0.77 - 1.60) | (1.00 - 2.24) | (0.80 - 1.32) | (0.88 - 1.47) | (0.50 - 0.77) | (1.11 - 1.65) |
| Dabat | 2015 | 0.51** | 0.60* | 1.00 | 0.88 | 0.96 | 1.23 | 0.72 | 0.98 | 0.45*** | 1.13 |
|  |  | (0.29 - 0.89) | (0.33 - 1.07) | (0.51 - 1.99) | (0.43 - 1.79) | (0.56 - 1.66) | (0.69 - 2.18) | (0.48 - 1.09) | (0.67 - 1.42) | (0.31 - 0.65) | (0.83 - 1.53) |
| Arba Minch | 2010 | 0.63*** | 0.66*** | 1.10 | 1.03 | 1.19 | 0.97 | 0.99 | 0.81 | 0.49*** | 0.78* |
|  |  | (0.48 - 0.84) | (0.48 - 0.90) | (0.73 - 1.68) | (0.68 - 1.58) | (0.85 - 1.66) | (0.65 - 1.45) | (0.78 - 1.25) | (0.63 - 1.05) | (0.39 - 0.61) | (0.61 - 1.00) |
| Arba Minch | 2015 | 0.52** | 0.66 | 1.25 | 1.68* | 0.93 | 0.83 | 0.66* | 0.57** | 0.73* | 0.93 |
|  |  | (0.30 - 0.90) | (0.38 - 1.14) | (0.68 - 2.31) | (0.96 - 2.94) | (0.52 - 1.64) | (0.42 - 1.62) | (0.44 - 1.01) | (0.36 - 0.90) | (0.53 - 1.01) | (0.63 - 1.37) |
| Navrongo | 1990 | 3.65*** | 4.18*** | 5.54*** | 5.27*** | 2.33*** | 3.83*** | 4.75*** | 3.60*** | 2.00*** | 3.53*** |
|  |  | (2.79 - 4.80) | (3.12 - 5.60) | (3.59 - 8.56) | (3.38 - 8.24) | (1.48 - 3.66) | (2.45 - 6.00) | (3.76 - 6.00) | (2.82 - 4.61) | (1.61 - 2.50) | (2.88 - 4.34) |
| Navrongo | 1995 | 3.09*** | 3.30*** | 3.90*** | 3.01*** | 2.26*** | 2.65*** | 3.54*** | 2.62*** | 1.75*** | 2.31*** |
|  |  | (2.51 - 3.81) | (2.63 - 4.16) | (2.69 - 5.66) | (2.05 - 4.40) | (1.67 - 3.08) | (1.85 - 3.80) | (2.91 - 4.32) | (2.13 - 3.23) | (1.49 - 2.05) | (1.96 - 2.71) |
| Navrongo | 2000 | 2.17*** | 2.34*** | 3.14*** | 2.20*** | 2.03*** | 2.45*** | 3.33*** | 2.22*** | 1.64*** | 1.99*** |
|  |  | (1.74 - 2.70) | (1.84 - 2.98) | (2.15 - 4.59) | (1.48 - 3.27) | (1.50 - 2.75) | (1.71 - 3.51) | (2.74 - 4.06) | (1.80 - 2.74) | (1.40 - 1.92) | (1.70 - 2.33) |
| Navrongo | 2005 | 1.37** | 1.54*** | 1.88*** | 1.41 | 1.26 | 1.44* | 3.07*** | 1.50*** | 1.58*** | 1.40*** |
|  |  | (1.08 - 1.73) | (1.19 - 1.99) | (1.25 - 2.81) | (0.92 - 2.15) | (0.92 - 1.74) | (0.99 - 2.11) | (2.52 - 3.74) | (1.21 - 1.87) | (1.35 - 1.85) | (1.20 - 1.65) |
| Navrongo | 2010 | 0.86 | 0.89 | 1.45* | 0.86 | 1.18 | 1.03 | 3.39*** | 1.29** | 1.51*** | 1.26*** |
|  |  | (0.66 - 1.11) | (0.67 - 1.18) | (0.96 - 2.21) | (0.54 - 1.38) | (0.86 - 1.62) | (0.70 - 1.52) | (2.79 - 4.13) | (1.04 - 1.61) | (1.29 - 1.77) | (1.07 - 1.48) |
| Kintampo | 2005 | 0.96 | 1.26* | 1.79*** | 1.65** | 1.80*** | 2.04*** | 1.95*** | 1.40*** | 0.80** | 0.74*** |
|  |  | (0.76 - 1.22) | (0.98 - 1.62) | (1.21 - 2.64) | (1.11 - 2.44) | (1.33 - 2.44) | (1.43 - 2.91) | (1.59 - 2.39) | (1.13 - 1.75) | (0.67 - 0.95) | (0.61 - 0.90) |
| Kintampo | 2010 | 0.91 | 0.93 | 1.23 | 1.06 | 1.45** | 1.42** | 1.90*** | 1.15 | 0.84** | 0.73*** |
|  |  | (0.73 - 1.14) | (0.73 - 1.19) | (0.84 - 1.82) | (0.71 - 1.57) | (1.08 - 1.95) | (1.00 - 2.03) | (1.56 - 2.31) | (0.92 - 1.42) | (0.72 - 0.99) | (0.61 - 0.87) |
| Dodowa | 2005 | 0.75** | 0.82 | 1.32 | 1.20 | 1.36* | 2.16*** | 2.32*** | 2.04*** | 0.97 | 1.01 |
|  |  | (0.58 - 0.98) | (0.62 - 1.08) | (0.87 - 1.99) | (0.79 - 1.81) | (0.99 - 1.86) | (1.52 - 3.07) | (1.89 - 2.84) | (1.65 - 2.53) | (0.82 - 1.16) | (0.85 - 1.20) |
| Dodowa | 2010 | 0.55*** | 0.72* | 1.30 | 1.21 | 1.05 | 1.54** | 1.66*** | 1.45*** | 0.90 | 0.91 |
|  |  | (0.39 - 0.77) | (0.50 - 1.02) | (0.81 - 2.06) | (0.76 - 1.94) | (0.72 - 1.52) | (1.04 - 2.28) | (1.32 - 2.09) | (1.14 - 1.83) | (0.73 - 1.10) | (0.74 - 1.11) |
| Farafenni | 1990 | 4.20*** | 5.42*** | 5.61*** | 5.83*** | 1.45 | 1.22 | 1.34* | 0.93 | 0.65*** | 0.95 |
|  |  | (3.31 - 5.34) | (4.22 - 6.98) | (3.68 - 8.57) | (3.84 - 8.86) | (0.89 - 2.39) | (0.65 - 2.29) | (0.97 - 1.85) | (0.65 - 1.33) | (0.47 - 0.89) | (0.70 - 1.29) |
| Farafenni | 1995 | 4.83*** | 4.47*** | 5.30*** | 4.23*** | 1.43 | 3.14*** | 2.45*** | 1.69*** | 1.48*** | 1.59*** |
|  |  | (3.81 - 6.12) | (3.42 - 5.84) | (3.47 - 8.10) | (2.73 - 6.56) | (0.86 - 2.37) | (2.00 - 4.93) | (1.88 - 3.21) | (1.26 - 2.27) | (1.18 - 1.85) | (1.25 - 2.01) |
| Farafenni | 2000 | 2.05*** | 2.76*** | 2.14*** | 1.97*** | 1.32 | 2.22*** | 2.39*** | 1.93*** | 1.58*** | 1.48*** |
|  |  | (1.60 - 2.63) | (2.12 - 3.58) | (1.36 - 3.37) | (1.24 - 3.13) | (0.87 - 2.01) | (1.48 - 3.34) | (1.89 - 3.02) | (1.50 - 2.49) | (1.30 - 1.92) | (1.20 - 1.83) |
| Farafenni | 2005 | 0.99 | 1.10 | 1.44 | 1.40 | 1.10 | 1.71*** | 1.91*** | 1.49*** | 1.30*** | 1.33*** |
|  |  | (0.76 - 1.29) | (0.83 - 1.47) | (0.91 - 2.26) | (0.88 - 2.21) | (0.74 - 1.63) | (1.16 - 2.53) | (1.53 - 2.40) | (1.17 - 1.90) | (1.08 - 1.57) | (1.09 - 1.62) |
| Farafenni | 2010 | 0.79* | 0.82 | 1.23 | 1.08 | 0.82 | 1.34 | 1.57*** | 1.34** | 1.13 | 1.20* |
|  |  | (0.60 - 1.03) | (0.61 - 1.11) | (0.77 - 1.94) | (0.68 - 1.73) | (0.54 - 1.24) | (0.90 - 2.01) | (1.25 - 1.98) | (1.05 - 1.70) | (0.94 - 1.37) | (0.99 - 1.47) |
| Farafenni | 2015 | 0.55** | 0.62 | 1.29 | 0.97 | 0.76 | 0.95 | 1.41* | 1.14 | 0.73* | 0.98 |
|  |  | (0.32 - 0.95) | (0.35 - 1.11) | (0.63 - 2.62) | (0.45 - 2.10) | (0.36 - 1.60) | (0.47 - 1.91) | (0.98 - 2.02) | (0.78 - 1.67) | (0.51 - 1.05) | (0.69 - 1.41) |
| Kombewa | 2010 | 1.09 | 1.37*** | 1.77*** | 1.24 | 1.88*** | 2.05*** | 2.59*** | 1.60*** | 0.94 | 0.88 |
|  |  | (0.87 - 1.37) | (1.08 - 1.75) | (1.21 - 2.60) | (0.83 - 1.84) | (1.39 - 2.53) | (1.44 - 2.91) | (2.11 - 3.17) | (1.29 - 1.99) | (0.79 - 1.11) | (0.74 - 1.04) |
| Kombewa | 2015 | 0.74 | 0.78 | 0.97 | 0.85 | 1.78*** | 1.63** | 2.17*** | 1.79*** | 1.02 | 0.84 |
|  |  | (0.51 - 1.07) | (0.52 - 1.14) | (0.58 - 1.62) | (0.50 - 1.45) | (1.24 - 2.55) | (1.07 - 2.48) | (1.70 - 2.76) | (1.39 - 2.30) | (0.83 - 1.26) | (0.68 - 1.05) |
| Karonga | 2000 | 1.09 | 1.43* | 2.41*** | 1.13 | 1.26 | 3.41*** | 4.43*** | 4.30*** | 0.79 | 1.06 |
|  |  | (0.71 - 1.66) | (0.93 - 2.20) | (1.32 - 4.39) | (0.52 - 2.44) | (0.70 - 2.27) | (2.10 - 5.55) | (3.33 - 5.88) | (3.22 - 5.74) | (0.54 - 1.16) | (0.76 - 1.47) |
| Karonga | 2005 | 1.08 | 1.19 | 1.20 | 0.90 | 1.08 | 1.71** | 2.82*** | 2.38*** | 0.70*** | 0.98 |
|  |  | (0.82 - 1.41) | (0.89 - 1.60) | (0.73 - 1.95) | (0.53 - 1.52) | (0.72 - 1.61) | (1.13 - 2.60) | (2.24 - 3.54) | (1.86 - 3.03) | (0.55 - 0.89) | (0.78 - 1.23) |
| Karonga | 2010 | 0.84 | 0.77 | 0.66 | 1.01 | 1.04 | 1.21 | 1.80*** | 1.00 | 0.60*** | 0.83 |
|  |  | (0.63 - 1.13) | (0.55 - 1.07) | (0.38 - 1.16) | (0.62 - 1.67) | (0.70 - 1.54) | (0.78 - 1.87) | (1.41 - 2.29) | (0.76 - 1.32) | (0.47 - 0.77) | (0.67 - 1.05) |
| Karonga | 2015 | 0.50*** | 0.43*** | 1.09 | 0.62 | 1.47* | 0.95 | 1.61*** | 0.96 | 0.86 | 0.57*** |
|  |  | (0.31 - 0.83) | (0.24 - 0.78) | (0.58 - 2.04) | (0.29 - 1.35) | (0.93 - 2.32) | (0.53 - 1.71) | (1.19 - 2.19) | (0.67 - 1.37) | (0.64 - 1.17) | (0.40 - 0.82) |
| Chokwe | 2010 | 1.64*** | 1.70*** | 1.57** | 1.08 | 2.53*** | 2.86*** | 4.25*** | 2.48*** | 1.23** | 0.97 |
|  |  | (1.32 - 2.04) | (1.34 - 2.16) | (1.05 - 2.35) | (0.71 - 1.65) | (1.86 - 3.43) | (2.02 - 4.05) | (3.47 - 5.20) | (2.01 - 3.06) | (1.02 - 1.48) | (0.80 - 1.16) |
| Chokwe | 2015 | 1.29 | 0.99 | 0.97 | 0.92 | 1.40 | 1.46 | 3.73*** | 1.86*** | 1.16 | 0.89 |
|  |  | (0.94 - 1.77) | (0.68 - 1.45) | (0.53 - 1.80) | (0.50 - 1.71) | (0.86 - 2.28) | (0.90 - 2.37) | (2.90 - 4.80) | (1.42 - 2.43) | (0.87 - 1.53) | (0.68 - 1.17) |
| Nahuche | 2010 | 7.92*** | 9.60*** | 4.98*** | 5.55*** | 1.70*** | 2.95*** | 1.35*** | 1.74*** | 0.87 | 1.08 |
|  |  | (6.55 - 9.58) | (7.78 - 11.83) | (3.48 - 7.12) | (3.88 - 7.94) | (1.26 - 2.31) | (2.09 - 4.16) | (1.09 - 1.67) | (1.40 - 2.17) | (0.73 - 1.04) | (0.89 - 1.31) |
| Bandafassi | 1990 | 4.89*** | 6.15*** | 5.51*** | 2.82*** | 2.23*** | 3.85*** | 2.12*** | 1.78*** | 1.55*** | 1.90*** |
|  |  | (3.75 - 6.38) | (4.67 - 8.09) | (3.39 - 8.97) | (1.58 - 5.02) | (1.38 - 3.60) | (2.35 - 6.29) | (1.53 - 2.94) | (1.26 - 2.53) | (1.17 - 2.05) | (1.45 - 2.48) |
| Bandafassi | 1995 | 4.85*** | 4.86*** | 2.61*** | 4.85*** | 2.09*** | 3.57*** | 2.28*** | 1.97*** | 1.58*** | 1.87*** |
|  |  | (3.75 - 6.28) | (3.65 - 6.47) | (1.46 - 4.65) | (3.01 - 7.82) | (1.29 - 3.39) | (2.19 - 5.81) | (1.68 - 3.09) | (1.42 - 2.73) | (1.22 - 2.06) | (1.45 - 2.41) |
| Bandafassi | 2000 | 3.41*** | 4.78*** | 4.16*** | 4.17*** | 1.73** | 3.88*** | 1.65*** | 1.57*** | 0.93 | 1.75*** |
|  |  | (2.61 - 4.47) | (3.63 - 6.28) | (2.56 - 6.74) | (2.58 - 6.74) | (1.06 - 2.82) | (2.46 - 6.12) | (1.19 - 2.28) | (1.12 - 2.20) | (0.69 - 1.26) | (1.36 - 2.26) |
| Bandafassi | 2005 | 3.19*** | 3.78*** | 2.71*** | 2.70*** | 1.58* | 3.02*** | 1.59*** | 1.54** | 1.02 | 1.33** |
|  |  | (2.45 - 4.15) | (2.85 - 5.01) | (1.61 - 4.58) | (1.60 - 4.56) | (0.97 - 2.58) | (1.88 - 4.84) | (1.14 - 2.20) | (1.10 - 2.15) | (0.77 - 1.33) | (1.01 - 1.74) |
| Bandafassi | 2010 | 1.82*** | 2.03*** | 3.49*** | 2.20*** | 0.99 | 2.15*** | 1.56*** | 1.79*** | 0.96 | 1.25 |
|  |  | (1.34 - 2.47) | (1.46 - 2.83) | (2.17 - 5.60) | (1.28 - 3.75) | (0.58 - 1.71) | (1.30 - 3.58) | (1.13 - 2.16) | (1.31 - 2.46) | (0.74 - 1.26) | (0.96 - 1.63) |
| Bandafassi | 2015 | 1.01 | 0.82 | 0.86 | 1.32 | 1.05 | 2.04** | 1.78*** | 1.32 | 1.07 | 1.74*** |
|  |  | (0.58 - 1.77) | (0.43 - 1.58) | (0.30 - 2.42) | (0.55 - 3.17) | (0.50 - 2.21) | (1.01 - 4.10) | (1.18 - 2.70) | (0.82 - 2.14) | (0.75 - 1.53) | (1.25 - 2.40) |
| Mlomp | 1990 | 2.07*** | 3.02*** | 1.86* | 1.55 | 1.10 | 0.97 | 1.65*** | 1.23 | 1.37** | 1.09 |
|  |  | (1.34 - 3.19) | (2.00 - 4.56) | (0.89 - 3.90) | (0.72 - 3.37) | (0.60 - 2.02) | (0.43 - 2.17) | (1.14 - 2.39) | (0.77 - 1.97) | (1.06 - 1.77) | (0.80 - 1.47) |
| Mlomp | 1995 | 2.04*** | 1.99*** | 2.34** | 1.44 | 1.65* | 0.83 | 1.80*** | 0.86 | 1.43*** | 1.05 |
|  |  | (1.29 - 3.23) | (1.23 - 3.24) | (1.18 - 4.64) | (0.64 - 3.27) | (1.00 - 2.73) | (0.37 - 1.87) | (1.26 - 2.57) | (0.51 - 1.46) | (1.12 - 1.82) | (0.78 - 1.41) |
| Mlomp | 2000 | 1.92*** | 1.74** | 3.71*** | 1.22 | 1.71** | 1.99** | 1.99*** | 0.85 | 1.05 | 1.12 |
|  |  | (1.22 - 3.03) | (1.04 - 2.93) | (2.06 - 6.69) | (0.51 - 2.91) | (1.06 - 2.77) | (1.14 - 3.46) | (1.42 - 2.77) | (0.51 - 1.40) | (0.81 - 1.35) | (0.85 - 1.48) |
| Mlomp | 2005 | 0.92 | 0.57 | 0.69 | 0.88 | 0.82 | 1.33 | 1.09 | 0.58* | 0.98 | 0.82 |
|  |  | (0.49 - 1.70) | (0.23 - 1.41) | (0.21 - 2.26) | (0.31 - 2.48) | (0.44 - 1.54) | (0.71 - 2.51) | (0.71 - 1.66) | (0.31 - 1.09) | (0.75 - 1.29) | (0.60 - 1.13) |
| Mlomp | 2010 | 0.16** | 0.58 | 0.62 | 0.00 | 0.69 | 0.21** | 1.08 | 0.84 | 1.36** | 0.68** |
|  |  | (0.04 - 0.66) | (0.25 - 1.33) | (0.19 - 2.03) | (0.00 - .) | (0.35 - 1.36) | (0.05 - 0.86) | (0.73 - 1.59) | (0.50 - 1.41) | (1.06 - 1.75) | (0.48 - 0.94) |
| Mlomp | 2015 | 0.40 | 0.00 | 0.00 | 1.73 | 0.71 | 0.55 | 0.87 | 0.67 | 1.05 | 0.39*** |
|  |  | (0.10 - 1.63) | (0.00 - .) | (0.00 - .) | (0.53 - 5.64) | (0.26 - 1.97) | (0.13 - 2.27) | (0.46 - 1.66) | (0.27 - 1.65) | (0.69 - 1.59) | (0.20 - 0.76) |
| Niakhar | 1990 | 4.60*** | 5.22*** | 3.70*** | 2.95*** | 2.20*** | 2.04*** | 1.48*** | 1.43** | 1.01 | 1.31*** |
|  |  | (3.70 - 5.71) | (4.13 - 6.60) | (2.45 - 5.57) | (1.92 - 4.54) | (1.51 - 3.21) | (1.30 - 3.21) | (1.14 - 1.94) | (1.08 - 1.88) | (0.82 - 1.25) | (1.07 - 1.61) |
| Niakhar | 1995 | 5.97*** | 6.71*** | 6.07*** | 5.36*** | 2.54*** | 2.42*** | 1.68*** | 1.56*** | 1.29*** | 1.23** |
|  |  | (4.85 - 7.35) | (5.34 - 8.44) | (4.13 - 8.92) | (3.62 - 7.93) | (1.79 - 3.60) | (1.59 - 3.68) | (1.30 - 2.16) | (1.20 - 2.03) | (1.07 - 1.57) | (1.01 - 1.51) |
| Niakhar | 2000 | 3.33*** | 3.23*** | 2.73*** | 3.12*** | 1.20 | 2.05*** | 1.45*** | 1.22 | 1.12 | 1.18* |
|  |  | (2.67 - 4.16) | (2.52 - 4.14) | (1.78 - 4.18) | (2.05 - 4.74) | (0.81 - 1.77) | (1.35 - 3.12) | (1.12 - 1.88) | (0.92 - 1.61) | (0.91 - 1.36) | (0.97 - 1.45) |
| Niakhar | 2005 | 1.53*** | 2.04*** | 2.07*** | 1.04 | 1.20 | 1.61** | 1.58*** | 1.43*** | 1.15 | 1.08 |
|  |  | (1.20 - 1.97) | (1.58 - 2.64) | (1.34 - 3.20) | (0.62 - 1.74) | (0.83 - 1.74) | (1.05 - 2.44) | (1.23 - 2.02) | (1.10 - 1.86) | (0.95 - 1.40) | (0.89 - 1.33) |
| Niakhar (Reference category) | 2010 | ~ | ~ | ~ | ~ | ~ | ~ | ~ | ~ |  |  |
|  |  | ~ | ~ | ~ | ~ | ~ | ~ | ~ | ~ |  |  |
| Niakhar | 2015 | 0.69* | 0.63** | 0.75 | 0.48* | 0.71 | 0.65 | 1.28 | 0.70* | 0.71** | 1.13 |
|  |  | (0.47 - 1.02) | (0.40 - 1.00) | (0.38 - 1.48) | (0.21 - 1.09) | (0.40 - 1.27) | (0.33 - 1.31) | (0.94 - 1.75) | (0.46 - 1.05) | (0.54 - 0.95) | (0.88 - 1.46) |
| Ifakara Rural | 1995 | 1.89*** | 2.23*** | 1.78** | 2.44*** | 2.16*** | 3.38*** | 2.75*** | 2.10*** | 0.99 | 1.31** |
|  |  | (1.47 - 2.43) | (1.72 - 2.91) | (1.13 - 2.83) | (1.58 - 3.76) | (1.53 - 3.05) | (2.31 - 4.95) | (2.20 - 3.44) | (1.65 - 2.69) | (0.81 - 1.21) | (1.06 - 1.62) |
| Ifakara Rural | 2000 | 1.90*** | 2.53*** | 1.62** | 1.63** | 1.60*** | 3.17*** | 2.18*** | 1.90*** | 0.94 | 1.15 |
|  |  | (1.53 - 2.36) | (2.01 - 3.20) | (1.07 - 2.45) | (1.08 - 2.46) | (1.16 - 2.20) | (2.23 - 4.51) | (1.77 - 2.69) | (1.52 - 2.38) | (0.79 - 1.12) | (0.95 - 1.38) |
| Ifakara Rural | 2005 | 1.32** | 1.41*** | 1.60** | 1.26 | 1.22 | 2.31*** | 1.81*** | 1.81*** | 0.79*** | 0.93 |
|  |  | (1.05 - 1.65) | (1.11 - 1.80) | (1.07 - 2.39) | (0.83 - 1.90) | (0.88 - 1.68) | (1.62 - 3.29) | (1.47 - 2.22) | (1.46 - 2.25) | (0.66 - 0.95) | (0.77 - 1.11) |
| Ifakara Rural | 2010 | 0.94 | 1.00 | 1.15 | 0.83 | 0.95 | 1.43** | 1.27** | 1.27** | 0.70*** | 0.89 |
|  |  | (0.75 - 1.18) | (0.78 - 1.28) | (0.77 - 1.71) | (0.55 - 1.25) | (0.69 - 1.31) | (1.00 - 2.05) | (1.04 - 1.56) | (1.03 - 1.58) | (0.59 - 0.83) | (0.74 - 1.06) |
| Rufiji | 1995 | 1.96*** | 1.74*** | 2.63*** | 3.12*** | 1.72** | 5.44*** | 3.55*** | 3.44*** | 1.05 | 1.16 |
|  |  | (1.41 - 2.74) | (1.19 - 2.55) | (1.55 - 4.46) | (1.85 - 5.26) | (1.03 - 2.87) | (3.57 - 8.30) | (2.71 - 4.63) | (2.61 - 4.52) | (0.83 - 1.33) | (0.91 - 1.49) |
| Rufiji | 2000 | 1.49*** | 1.88*** | 2.10*** | 1.75*** | 1.79*** | 4.03*** | 2.21*** | 1.99*** | 0.74*** | 0.92 |
|  |  | (1.19 - 1.86) | (1.48 - 2.38) | (1.42 - 3.11) | (1.16 - 2.62) | (1.30 - 2.46) | (2.84 - 5.73) | (1.79 - 2.72) | (1.59 - 2.48) | (0.62 - 0.87) | (0.77 - 1.09) |
| Rufiji | 2005 | 1.59*** | 1.64*** | 1.73*** | 1.34 | 1.13 | 2.27*** | 1.71*** | 1.67*** | 0.67*** | 0.71*** |
|  |  | (1.27 - 1.98) | (1.29 - 2.09) | (1.16 - 2.58) | (0.88 - 2.03) | (0.80 - 1.58) | (1.57 - 3.28) | (1.38 - 2.12) | (1.34 - 2.08) | (0.56 - 0.79) | (0.60 - 0.85) |
| Rufiji | 2010 | 0.97 | 0.92 | 1.23 | 0.89 | 0.87 | 1.08 | 1.19 | 1.08 | 0.65*** | 0.58*** |
|  |  | (0.77 - 1.23) | (0.71 - 1.20) | (0.82 - 1.85) | (0.58 - 1.37) | (0.62 - 1.22) | (0.73 - 1.60) | (0.96 - 1.48) | (0.86 - 1.35) | (0.55 - 0.77) | (0.49 - 0.69) |
| Magu | 1990 | 2.61*** | 1.68 | 0.70 | 0.65 | 1.82 | 3.34*** | 3.57*** | 3.04*** | ~ | ~ |
|  |  | (1.47 - 4.63) | (0.78 - 3.63) | (0.10 - 5.13) | (0.09 - 4.77) | (0.66 - 5.02) | (1.41 - 7.91) | (2.15 - 5.95) | (1.71 - 5.42) | ~ | ~ |
| Magu | 1995 | 2.72*** | 2.61*** | 2.51*** | 2.47*** | 2.49*** | 3.79*** | 3.44*** | 2.79*** | 0.97 | 1.65*** |
|  |  | (2.11 - 3.50) | (1.97 - 3.45) | (1.57 - 4.01) | (1.55 - 3.94) | (1.71 - 3.61) | (2.54 - 5.66) | (2.71 - 4.36) | (2.15 - 3.61) | (0.74 - 1.27) | (1.29 - 2.10) |
| Magu | 2000 | 2.26*** | 1.92*** | 2.32*** | 1.58* | 2.16*** | 3.63*** | 2.99*** | 2.74*** | 1.20 | 1.23 |
|  |  | (1.74 - 2.92) | (1.44 - 2.57) | (1.47 - 3.68) | (0.96 - 2.60) | (1.49 - 3.13) | (2.46 - 5.36) | (2.36 - 3.79) | (2.14 - 3.51) | (0.94 - 1.53) | (0.95 - 1.58) |
| Magu | 2005 | 1.34** | 1.91*** | 1.92*** | 1.18 | 2.37*** | 2.35*** | 2.54*** | 1.83*** | 1.11 | 1.09 |
|  |  | (1.01 - 1.78) | (1.44 - 2.55) | (1.20 - 3.07) | (0.70 - 1.99) | (1.66 - 3.38) | (1.55 - 3.58) | (2.01 - 3.22) | (1.41 - 2.37) | (0.88 - 1.42) | (0.85 - 1.40) |
| Magu | 2010 | 1.01 | 1.12 | 2.07*** | 0.77 | 1.90*** | 1.28 | 1.33* | 1.00 | 0.96 | 1.00 |
|  |  | (0.70 - 1.45) | (0.76 - 1.65) | (1.26 - 3.42) | (0.40 - 1.50) | (1.25 - 2.90) | (0.74 - 2.20) | (0.98 - 1.80) | (0.71 - 1.40) | (0.72 - 1.28) | (0.74 - 1.34) |
| Iganga/Mayuge | 2005 | 1.94*** | 2.00*** | 1.56** | 1.38 | 1.59*** | 2.22*** | 2.34*** | 1.64*** | 1.02 | 1.13 |
|  |  | (1.55 - 2.42) | (1.57 - 2.54) | (1.03 - 2.36) | (0.90 - 2.09) | (1.13 - 2.23) | (1.53 - 3.23) | (1.88 - 2.91) | (1.28 - 2.09) | (0.83 - 1.25) | (0.92 - 1.39) |
| Iganga/Mayuge | 2010 | 1.46*** | 1.71*** | 1.60** | 0.90 | 1.30 | 1.34 | 1.34** | 0.97 | 0.81** | 1.15 |
|  |  | (1.17 - 1.83) | (1.34 - 2.18) | (1.07 - 2.39) | (0.58 - 1.39) | (0.93 - 1.82) | (0.91 - 1.97) | (1.06 - 1.68) | (0.76 - 1.25) | (0.66 - 1.00) | (0.95 - 1.41) |
| Iganga/Mayuge | 2015 | 1.11 | 1.45* | 1.00 | 0.72 | 1.05 | 1.05 | 1.14 | 1.00 | 1.07 | 1.38** |
|  |  | (0.76 - 1.60) | (1.00 - 2.10) | (0.52 - 1.91) | (0.35 - 1.46) | (0.62 - 1.77) | (0.58 - 1.89) | (0.80 - 1.62) | (0.68 - 1.47) | (0.78 - 1.48) | (1.02 - 1.87) |
| Agincourt | 1990 | 0.58*** | 0.63** | 0.78 | 0.57* | 0.96 | 0.85 | 1.60*** | 1.02 | 0.83 | 0.73** |
|  |  | (0.40 - 0.85) | (0.42 - 0.94) | (0.43 - 1.42) | (0.29 - 1.10) | (0.62 - 1.49) | (0.51 - 1.43) | (1.22 - 2.09) | (0.75 - 1.39) | (0.64 - 1.07) | (0.56 - 0.95) |
| Agincourt | 1995 | 0.64*** | 0.76* | 0.71 | 0.58** | 1.11 | 1.38* | 2.07*** | 1.12 | 0.93 | 0.80** |
|  |  | (0.48 - 0.85) | (0.56 - 1.02) | (0.44 - 1.14) | (0.35 - 0.94) | (0.80 - 1.55) | (0.95 - 2.02) | (1.67 - 2.56) | (0.88 - 1.42) | (0.77 - 1.13) | (0.66 - 0.96) |
| Agincourt | 2000 | 1.13 | 1.09 | 0.83 | 0.78 | 2.14*** | 3.35*** | 3.65*** | 2.65*** | 1.15 | 0.76*** |
|  |  | (0.88 - 1.46) | (0.83 - 1.44) | (0.53 - 1.31) | (0.49 - 1.24) | (1.59 - 2.88) | (2.37 - 4.73) | (2.99 - 4.46) | (2.15 - 3.28) | (0.96 - 1.37) | (0.63 - 0.91) |
| Agincourt | 2005 | 0.88 | 0.98 | 1.25 | 1.14 | 2.19*** | 3.52*** | 4.88*** | 3.53*** | 1.32*** | 0.83** |
|  |  | (0.68 - 1.14) | (0.74 - 1.30) | (0.81 - 1.91) | (0.74 - 1.75) | (1.64 - 2.94) | (2.50 - 4.95) | (4.02 - 5.93) | (2.88 - 4.34) | (1.12 - 1.57) | (0.69 - 0.99) |
| Agincourt | 2010 | 0.53*** | 0.41*** | 1.12 | 0.46*** | 1.33* | 2.13*** | 3.01*** | 2.18*** | 1.16* | 0.80** |
|  |  | (0.40 - 0.70) | (0.29 - 0.57) | (0.74 - 1.71) | (0.28 - 0.76) | (0.99 - 1.79) | (1.51 - 3.01) | (2.47 - 3.66) | (1.77 - 2.69) | (0.98 - 1.38) | (0.67 - 0.95) |
| Agincourt | 2015 | 0.41*** | 0.22*** | 1.14 | 0.56* | 1.13 | 1.71*** | 2.45*** | 1.78*** | 1.20* | 0.89 |
|  |  | (0.27 - 0.61) | (0.12 - 0.38) | (0.69 - 1.87) | (0.31 - 1.02) | (0.80 - 1.59) | (1.17 - 2.49) | (1.98 - 3.02) | (1.42 - 2.24) | (0.99 - 1.46) | (0.73 - 1.09) |
| Dimamo | 1995 | 0.77 | 0.50 | 0.84 | 0.66 | 1.01 | 1.91** | 2.50*** | 1.12 | 0.95 | 0.69* |
|  |  | (0.39 - 1.53) | (0.20 - 1.23) | (0.30 - 2.37) | (0.20 - 2.15) | (0.50 - 2.04) | (1.02 - 3.60) | (1.78 - 3.51) | (0.70 - 1.79) | (0.64 - 1.41) | (0.47 - 1.02) |
| Dimamo | 2000 | 0.25** | 0.61 | 0.19 | 0.37 | 1.56 | 2.40*** | 3.35*** | 1.68*** | 1.30 | 0.92 |
|  |  | (0.08 - 0.80) | (0.27 - 1.40) | (0.03 - 1.39) | (0.09 - 1.54) | (0.92 - 2.67) | (1.40 - 4.14) | (2.54 - 4.43) | (1.17 - 2.40) | (0.94 - 1.78) | (0.68 - 1.26) |
| Dimamo | 2005 | 0.55 | 0.78 | 0.45 | 0.63 | 1.35 | 2.63*** | 3.17*** | 2.63*** | 0.93 | 0.85 |
|  |  | (0.24 - 1.26) | (0.38 - 1.61) | (0.11 - 1.89) | (0.19 - 2.06) | (0.78 - 2.33) | (1.56 - 4.42) | (2.40 - 4.19) | (1.94 - 3.56) | (0.66 - 1.32) | (0.62 - 1.17) |
| Dimamo | 2010 | 0.32*** | 0.31*** | 0.81 | 0.61 | 1.37* | 1.85*** | 2.61*** | 2.15*** | 1.28** | 0.75*** |
|  |  | (0.19 - 0.53) | (0.17 - 0.54) | (0.44 - 1.49) | (0.32 - 1.19) | (0.96 - 1.94) | (1.25 - 2.74) | (2.10 - 3.24) | (1.71 - 2.71) | (1.06 - 1.55) | (0.62 - 0.93) |
| Dimamo | 2015 | 0.24*** | 0.22*** | 0.22** | 0.51 | 1.23 | 1.55* | 2.06*** | 1.59*** | 1.09 | 0.83 |
|  |  | (0.10 - 0.58) | (0.08 - 0.60) | (0.05 - 0.91) | (0.20 - 1.32) | (0.79 - 1.93) | (0.95 - 2.53) | (1.59 - 2.67) | (1.20 - 2.10) | (0.86 - 1.40) | (0.65 - 1.06) |
| Africa Health  Research Institute | 2000 | 1.24* | 1.45*** | 1.53** | 1.48* | 3.96*** | 7.48*** | 8.13*** | 4.97*** | 1.65*** | 1.17* |
|  |  | (0.97 - 1.58) | (1.11 - 1.88) | (1.01 - 2.30) | (0.99 - 2.23) | (2.96 - 5.30) | (5.35 - 10.46) | (6.69 - 9.88) | (4.05 - 6.11) | (1.39 - 1.96) | (0.99 - 1.39) |
| Africa Health  Research Institute | 2005 | 0.93 | 0.88 | 0.99 | 1.21 | 3.39*** | 4.98*** | 6.40*** | 4.12*** | 1.61*** | 1.23** |
|  |  | (0.72 - 1.20) | (0.66 - 1.17) | (0.64 - 1.55) | (0.79 - 1.86) | (2.53 - 4.55) | (3.55 - 6.98) | (5.26 - 7.79) | (3.35 - 5.06) | (1.36 - 1.92) | (1.04 - 1.45) |
| Africa Health  Research Institute | 2010 | 0.40*** | 0.50*** | 1.12 | 0.66 | 2.14*** | 2.54*** | 3.89*** | 2.06*** | 1.46*** | 1.00 |
|  |  | (0.29 - 0.56) | (0.36 - 0.70) | (0.73 - 1.74) | (0.41 - 1.08) | (1.58 - 2.89) | (1.79 - 3.61) | (3.18 - 4.75) | (1.66 - 2.55) | (1.23 - 1.75) | (0.84 - 1.19) |
| Africa Health  Research Institute | 2015 | 0.17*** | 0.38*** | 0.73 | 0.47** | 1.26 | 1.60** | 2.59*** | 1.85*** | 1.37*** | 0.87 |
|  |  | (0.08 - 0.34) | (0.22 - 0.66) | (0.39 - 1.37) | (0.22 - 0.98) | (0.85 - 1.87) | (1.05 - 2.46) | (2.05 - 3.28) | (1.45 - 2.35) | (1.10 - 1.70) | (0.70 - 1.08) |
| **Migration status variables** |  |  |  |  |  |  |  |  |  |  |  |
| Permanent resident (Reference category) |  | ~ | ~ | ~ | ~ | ~ | ~ | ~ | ~ | ~ | ~ |
|  |  | ~ | ~ | ~ | ~ | ~ | ~ | ~ | ~ | ~ | ~ |
| <2y in-migrant |  | 0.96 | 0.95 | 1.03 | 1.10 | 0.93 | 1.12*** | 1.24*** | 1.51*** | 1.27*** | 1.35*** |
|  |  | (0.90 - 1.03) | (0.88 - 1.02) | (0.91 - 1.16) | (0.97 - 1.24) | (0.84 - 1.03) | (1.04 - 1.21) | (1.17 - 1.31) | (1.43 - 1.61) | (1.16 - 1.38) | (1.25 - 1.46) |
| 2-5y in-migrant |  | 0.77*** | 0.91 | 1.13** | 1.12* | 1.01 | 1.04 | 1.11*** | 1.34*** | 1.12*** | 1.36*** |
|  |  | (0.67 - 0.88) | (0.80 - 1.04) | (1.01 - 1.26) | (1.00 - 1.26) | (0.92 - 1.12) | (0.96 - 1.12) | (1.05 - 1.17) | (1.26 - 1.42) | (1.04 - 1.22) | (1.26 - 1.46) |
| 5y+ in-migrant |  | ~ | ~ | 1.05 | 1.06 | 1.03 | 1.00 | 1.12*** | 1.29*** | 1.17*** | 1.19*** |
|  |  | ~ | ~ | (0.90 - 1.22) | (0.89 - 1.25) | (0.91 - 1.17) | (0.90 - 1.11) | (1.05 - 1.19) | (1.21 - 1.38) | (1.07 - 1.27) | (1.09 - 1.29) |
| <2y return migrant |  | ~ | ~ | 1.47*** | 1.23* | 1.50*** | 1.70*** | 1.43*** | 1.86*** | 1.45*** | 1.53*** |
|  |  | ~ | ~ | (1.19 - 1.83) | (0.97 - 1.56) | (1.32 - 1.71) | (1.52 - 1.91) | (1.31 - 1.57) | (1.69 - 2.06) | (1.24 - 1.70) | (1.33 - 1.74) |
| 2-5y return migrant |  | ~ | ~ | ~ | ~ | 1.22** | 1.35*** | 1.31*** | 1.46*** | 1.08 | 1.22*** |
|  |  | ~ | ~ | ~ | ~ | (1.05 - 1.41) | (1.18 - 1.54) | (1.20 - 1.42) | (1.32 - 1.61) | (0.93 - 1.26) | (1.07 - 1.39) |
|  |  |  |  |  |  |  |  |  |  |  |  |
| 5y+ return migrant |  | ~ | ~ | ~ | ~ | 1.32*** | 1.04 | 1.13** | 1.26*** | 1.02 | 1.10 |
|  |  | ~ | ~ |  |  | (1∙08 - 1∙60) | (0∙84 - 1∙27) | (1∙02 - 1∙25) | (1∙12 - 1∙42) | (0∙86 - 1∙19) | (0∙95 - 1∙28) |
| Observations |  | 792 934 | 785 313 | 1 399 245 | 1 409 654 | 1 334 384 | 1 621 498 | 984 374 | 1 148 980 | 193 327 | 256 661 |
| Log Likelihood |  | -171 727 | -157 488 | -65 443 | -54 817 | -81 568 | -93 144 | -264 967 | -218 059 | -189 880 | -188 433 |
| Number of people |  | 483 625 | 479 679 | 668 119 | 669 505 | 587 997 | 693 789 | 388 013 | 438 806 | 86 436 | 108 652 |
| Time at risk |  | 1 109 000 | 1 090 000 | 2 557 000 | 2 458 000 | 2 360 000 | 2 407 000 | 2 022 000 | 2 414 000 | 458 207 | 593 125 |
| Deaths |  | 14 036 | 12 917 | 5 311 | 4 480 | 6 901 | 7 888 | 24 236 | 19 531 | 19 124 | 18 542 |

Confidence intervals in parentheses

*** p<0.01 ** p<0.05 * p<0.1

**Supplementary Material: Appendix 2: Cox proportional hazards models: Urban HDSS**

| **Variables** |  | **Age 15-29 mortality males** | **Age 15-29 mortality females** | **Age 30-59 mortality males** | **Age 30-59 mortality females** | **Age 60-79 mortality males** | **Age 60-79 mortality females** |
| --- | --- | --- | --- | --- | --- | --- | --- |
| **Centre period variables** |  |  |  |  |  |  |  |
| Ouagadougou | 2005 | 0.53* | 1.81** | 0.82 | 1.38* | 0.87 | 0.89 |
|  |  | (0.27 - 1.06) | (1.11 - 2.94) | (0.58 - 1.15) | (0.94 - 2.02) | (0.59 - 1.28) | (0.55 - 1.44) |
| Ouagadougou (Reference category) | 2010 | ~ | ~ | ~ | ~ | ~ | ~ |
|  |  | ~ | ~ | ~ | ~ | ~ | ~ |
| Ouagadougou | 2015 | 1.26 | 1.55* | 1.08 | 1.20 | 0.98 | 1.09 |
|  |  | (0.75 - 2.10) | (0.95 - 2.55) | (0.84 - 1.40) | (0.86 - 1.67) | (0.71 - 1.36) | (0.74 - 1.59) |
| Harar Urban | 2010 | 0.92 | 0.73 | 1.25* | 1.50*** | 0.96 | 0.89 |
|  |  | (0.52 - 1.63) | (0.37 - 1.42) | (0.97 - 1.61) | (1.11 - 2.03) | (0.70 - 1.33) | (0.63 - 1.25) |
| Harar Urban | 2015 | 0.51** | 0.78 | 0.89 | 1.28* | 0.83 | 1.09 |
|  |  | (0.26 - 0.98) | (0.44 - 1.40) | (0.69 - 1.14) | (0.97 - 1.69) | (0.63 - 1.10) | (0.83 - 1.44) |
| Nairobi | 2000 | 2.98*** | 4.97*** | 2.54*** | 5.98*** | 0.88 | 1.33 |
|  |  | (2.20 - 4.04) | (3.62 - 6.83) | (2.14 - 3.02) | (4.79 - 7.45) | (0.58 - 1.35) | (0.81 - 2.18) |
| Nairobi | 2005 | 3.33*** | 3.72*** | 1.94*** | 3.61*** | 0.94 | 1.24 |
|  |  | (2.55 - 4.36) | (2.79 - 4.97) | (1.68 - 2.24) | (2.97 - 4.37) | (0.72 - 1.23) | (0.88 - 1.74) |
| Nairobi | 2010 | 3.75*** | 2.90*** | 1.68*** | 2.71*** | 0.79* | 1.26 |
|  |  | (2.84 - 4.96) | (2.12 - 3.96) | (1.45 - 1.95) | (2.22 - 3.32) | (0.60 - 1.03) | (0.91 - 1.74) |
| Nairobi | 2015 | 2.03*** | 1.73* | 1.16 | 1.67*** | 0.65 | 1.85** |
|  |  | (1.25 - 3.30) | (0.98 - 3.07) | (0.89 - 1.51) | (1.18 - 2.37) | (0.39 - 1.09) | (1.12 - 3.06) |
| **Migration status variables** |  |  |  |  |  |  |  |
| Permanent resident (Reference category) |  | ~ | ~ | ~ | ~ | ~ | ~ |
|  |  | ~ | ~ | ~ | ~ | ~ | ~ |
| <2y migrant |  | 0.62*** | 0.92 | 1.06 | 1.27*** | 0.85 | 1.15 |
|  |  | (0.51 - 0.77) | (0.73 - 1.15) | (0.92 - 1.22) | (1.06 - 1.52) | (0.59 - 1.24) | (0.81 - 1.65) |
| 2-5y migrant |  | 0.79** | 0.88 | 1.07 | 1.12 | 0.89 | 0.91 |
|  |  | (0.63 - 0.98) | (0.68 - 1.14) | (0.93 - 1.23) | (0.93 - 1.35) | (0.62 - 1.26) | (0.61 - 1.36) |
|  |  |  |  |  |  |  |  |
| 5y+ migrant |  | 0.89 | 0.85 | 0.97 | 1.18 | 0.77 | 0.99 |
|  |  | (0.64 - 1.24) | (0.57 - 1.28) | (0.80 - 1.17) | (0.94 - 1.49) | (0.44 - 1.34) | (0.55 - 1.78) |
| Observations |  | 23 939 | 245 337 | 212 953 | 132 569 | 12 773 | 11 628 |
| Log Likelihood |  | -6 560 | -5303 | -17 199 | -10282 | -3 976 | -3 072 |
| Number of people |  | 90 323 | 96 332 | 72 051 | 47 515 | 5 467 | 5 529 |
| Time at risk |  | 216 378 | 225 161 | 254 019 | 167 459 | 18 526 | 17 930 |
| Deaths |  | 693 | 559 | 1 925 | 1 207 | 589 | 458 |

Confidence intervals in parentheses

*** p<0.01 ** p<0.05 * p<0.1
